# Midbrain organoids—development and applications in Parkinson’s disease

**DOI:** 10.1093/oons/kvad009

**Published:** 2023-08-18

**Authors:** Hilary S Y Toh, Xin Yi Choo, Alfred Xuyang Sun

**Affiliations:** Neuroscience & Behavioural Disorders Programme, Duke-NUS Medical School, 8 College Road, Singapore; Neuroscience & Behavioural Disorders Programme, Duke-NUS Medical School, 8 College Road, Singapore; Neuroscience & Behavioural Disorders Programme, Duke-NUS Medical School, 8 College Road, Singapore; National Neuroscience Institute, 11 Jln Tan Tock Seng, Singapore

**Keywords:** Parkinson, brain organoid, degeneration, dopamine neuron, disease modelling

## Abstract

Human brain development is spatially and temporally complex. Insufficient access to human brain tissue and inadequacy of animal models has limited the study of brain development and neurodegenerative diseases. Recent advancements of brain organoid technology have created novel opportunities to model human-specific neurodevelopment and brain diseases. In this review, we discuss the use of brain organoids to model the midbrain and Parkinson’s disease. We critically evaluate the extent of recapitulation of PD pathology by organoids and discuss areas of future development that may lead to the model to become a next-generation, personalized therapeutic strategy for PD and beyond.

## INTRODUCTION

Human brain development is spatially and temporally complex. Numerous studies have characterized the brain’s anatomical structure, molecular features and electrophysiological functions. However, insufficient access to human brain tissue and inadequacy of animal models has limited the study of brain development and neurodegenerative diseases. Recent advancements of brain organoid technology have created novel opportunities to model human-specific neurodevelopment. Brain organoids can also aid translational research for intractable age-related neurological disorders, such as Parkinson’s disease (PD) and Alzheimer’s disease (AD), which together represent an increasing proportion of global disease burden [1].

**Figure 1:**
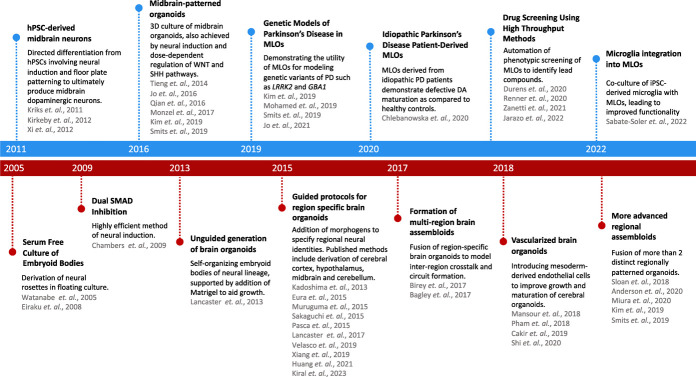
Timeline of key milestones in methods to derive midbrain dopaminergic neurons & MLOs (in blue). Timeline of key milestones in 2D neuronal and 3D organoid culture (in red)

In this review, we discuss the use of brain organoids to model the midbrain and its associated neurodegenerative disease: PD (Fig. 1). The hallmark of PD is the selective death of midbrain dopaminergic (mDA) neurons in the substantia nigra pars compacta, leading to the degeneration of the nigrostriatal pathway [2]. This manifests clinically as motor deficiencies including bradykinesia, rigidity, tremors and postural instability [3]. PD has been extensively studied using animal models; furthermore, the ability to produce human neurons from human pluripotent stem cells (hPSCs) has allowed PD mechanisms to be modelled in a human context. The generation of patient-derived induced pluripotent stem cells (iPSCs) has enabled the study of varied genetic susceptibilities and treatment responses and the evolution of personalized therapeutic strategies [4].

## GENERATING HUMAN DOPAMINERGIC NEURONAL MODELS

### Directed differentiation of dopaminergic neurons

Two-dimensional (2D) neuronal cultures have provided insights into midbrain development and the pathogenesis of neurodegenerative diseases. Early trials of neural differentiation from hPSCs involved co-culturing with stromal feeder cells to improve neural induction, facilitated by secreted factors termed as stromal cell-derived inducing activity (SDIA) [5]. Dual SMAD inhibition, a method developed by Chambers et al. [6] greatly increased the efficiency of neural induction. hPSCs were efficiently directed towards the neuroectodermal lineage by inhibiting transforming growth factor-β (TGF-β) as well as bone morphogenetic protein (BMP) signalling pathways [6, 7]. Most methods use a combination of SB431542 and Noggin or LDN-193189 to achieve dual SMAD inhibition, as this method simplified the neural induction process and expanded studies of stem cell differentiation into a variety of neural cell types [8–10].

Directed differentiation of hPSCs into mDA neurons typically involves neuralization by dual SMAD inhibition, followed by exposure to small molecules that pattern neural progenitors towards the midbrain lineage. These include morphogens like sonic hedgehog (SHH), activators of WNT signalling and FGF8 as observed in early neurodevelopmental studies that used mouse models to dissect mDA neuron development. Located at the midbrain–hindbrain boundary [11], the isthmic organizer expresses *Otx2* and *Gbx2,* which regulate *Wnt1* and *Fgf8* in the midbrain and hindbrain, respectively. This transcriptional network controls the anteroposterior patterning of the midbrain–hindbrain region. Specifically, *Wnt1* activates *Lmx1a*, which coordinates a network of transcription factors required for midbrain specification [12]. The other major signalling centre for midbrain patterning—the floor plate—is responsible for regulating ventrodorsal patterning. The floor plate is the most ventral region of the neural plate and expresses the morphogen SHH, which mediates midbrain neurogenesis by activating the *Foxa2* transcription factor. An SHH gradient is formed along the ventrodorsal axis, the ventral progenitors, exposed to the highest concentrations of SHH, are primed to become floor plate neural progenitors [13].

In 2011, Kriks et al. [14] proposed an improved protocol which incorporated CHIR99021, a potent activator of WNT signalling. The protocol utilized pumorphamine (an SHH agonist), FGF8 and CHIR99021 to mimic developmental processes and was able to generate higher yields of midbrain-patterned progenitors expressing key markers such as FOXA2 and LMX1A. Subsequent work by Kirkeby et al. and Xi et al. [15, 16] uncovered the temporal and dose-dependent effects of CHIR99021 on midbrain patterning. These 2D differentiation protocols form the basis for deriving midbrain-like organoids (MLOs) [14–18], discussed in detail in Section 1.2.

2D neuronal differentiation methods are effective for generating single neuronal types, and are thus widely used in preclinical studies of neuronal development and disease modelling. However, monolayer cultures are inherently limited in their ability to precisely model the *in vivo* conditions of the human brain. While striving for high purity yields of a particular cell type, they are unable to adequately model cell-to-cell communication between different neuronal subtypes or neuron–glia interactions. Moreover, cells *in vivo* are surrounded by extracellular matrices, whose stiffness is known to regulate cellular differentiation and axonal outgrowth [19, 20]. Overall, since 2D neuronal culture is unable to mimic the full range of biochemical and biophysical interactions *in vivo*, advanced culture conditions that reflect *in vivo* conditions more closely are needed, leading to the emergence of three-dimensional (3D) neuronal models.

### Generating three-dimensional brain organoids

Organoids are 3D cellular aggregates generated from PSCs, including both embryonic stem cells (ESCs) and induced-PSCs (iPSCs) as starting materials. The generation of brain organoid is achieved using two main methodologies: unguided and guided protocols. As the term suggests, unguided protocols do not use extrinsic factors to restrict cell fate. Hence, these methods typically produce more diverse cell types. In 2005, Watanabe et al. [21] pioneered an unguided method of culturing mouse ESC aggregates in serum-free suspension culture to generate neural embryoid bodies (EBs). The same group, led by Yoshiki Sasai, further optimized their method by culturing EBs in U-bottom plates to improve the uniformity of aggregated EBs [22]. Leveraging on the self-organizing capacity of PSCs, subsequent unguided protocols for cerebral organoids allowed for spontaneous morphogenesis, creating brain organoids that contain neural populations from multiple regions [23, 24].

Single-cell transcriptomic profiling of hPSC-derived cerebral organoids showed that they resemble the developing fetal brain and were able to recapitulate the temporal sequence of development seen *in vivo* [25]. This justified their use for understanding human-specific physiological and pathological processes. However, the heterogeneity of unguided protocols also generated considerable batch variation, which prevented systematic studies of specific brain regions.

In contrast, guided protocols typically utilize small molecules that mimic morphogen signalling during development to produce regionalized brain organoids. These include organoids of the cortex [26–28], thalamus [29, 30], hippocampus [31], hypothalamus [28, 32–34], midbrain [28, 35] and cerebellum [36] to name a few. In the following sections, we will be discussing MLOs and their applications in greater detail.

### Development of midbrain-like organoids

In the last decade, several groups have generated MLOs [28, 35, 37–41], using protocols that share some common features. First, uniform EBs are generated from hPSCs by aggregating cells in low-attachment plates. Next, as in the 2D differentiation protocols, the organoids undergo neural induction and patterning towards the midbrain lineage. In some protocols, extracellular matrices such as Matrigel are added to the culture to provide structural support for the growth of neuroepithelial buds. The patterning process takes between 8 and 14 days, following which MLOs are typically maintained on an orbital shaker to facilitate nutrient and oxygen exchange.

Our group published a protocol to robustly generate MLOs from hPSCs in 2016 [35]. The protocol involved simultaneously treating EBs with dual SMAD inhibition factors and morphogens, such as SHH, CHIR99021 and FGF8, to derive mesencephalic neural progenitors. The observation of many cells double-positive for FOXA2 and tyrosine hydroxylase (TH) in MLOs validated successful differentiation towards midbrain dopamine neurons. Of note, this study compared MLOs and 2D-differentiation midbrain neurons against the fetal midbrain and found MLOs to be more transcriptionally similar to the *in vivo* condition.

Besides hPSCs, neuroepithelial stem cells (NESCs) can also be used as the starting population to generate EBs, as demonstrated by Monzel et al. [37]. NESCs are able to self-renew and differentiate into neurons [42]. When treated with midbrain patterning factors that manipulate the WNT and SHH signalling pathways, the authors observed a large proportion of neurons positive for FOXA2, LMX1A and TH. After prolonged culturing, a small population of astrocytes positive for S100B and oligodendrocytes developed in MLOs. The sequential appearance of glia after neurons was also noted by Fiorenzano et al. [39] when they performed a time course analysis of MLO development over four months.

Interestingly, neuromelanin (NM) granules have also been detected in MLOs as a by-product of dopamine synthesis [35, 43]. This finding is remarkable as NM is uniquely found in primate brains and has not been reported in organoids derived from mouse PSCs or in rodent brains [44].

## BIOLOGICAL RELEVANCE OF MLOS

### Dopaminergic neuronal subtypes in rodents and humans

A comparison of published MLO generation protocols found that most MLOs contain multiple neuronal populations, including but not limited to dopaminergic, GABAergic and serotonergic neurons [45]. All MLO models so far have reported the expression of TH and dopamine transporter (DAT) as markers of mature mDA neurons. Immunohistochemical staining of neurotransmitter expression also revealed the presence of glutamatergic, serotonergic and GABAergic neurons in MLOs.

As mDA neurons are the focus of most research involving midbrain pathology, MLO protocols often characterize the dopaminergic neurons in greater detail. mDA neurons are usually grouped according to their anatomical location: the A9 group is found in the substantia nigra pars compacta (SNc), A10 group is located in the ventral tegmental area (VTA) and a smaller cluster of A8 neurons is found in the retro-rubral area. Advances in single-cell transcriptomic profiling have facilitated the identification of molecular subtypes of mDA neurons. Early work establishing the molecular differences between A9 and A10 dopaminergic neurons were based on mouse models. Poulin et al. [46] defined six molecular subtypes of mDA neurons, two in the SNc and four in the VTA region. This was validated by multiple groups using single-cell transcriptomics to characterize the developing mouse brain [47–49]. Based on existing data, dopaminergic neurons in the mouse midbrain can be broadly sorted into seven groups that express either *Sox6* or *Vglut2*. Subtype 1 (*Sox6^+^Aldh1a1^+^)* can be found in the SNc while subtype 2 (*Sox6^+^Aldh1a1*^−^) is mainly in the VTA. Subtypes 3 and 4 (*Vglut2^+^Aldh1a1*^−^) span both the substantia nigra pars lateralis and VTA regions. Subtype 5 (*Vglut2^+^Vgat*^+^) neurons border the SNc and VTA. Subtype 6 (*Vglut2^+^Aldh1a1^+^Otx2*^+^) is located in various nuclei that form the VTA, including the nucleus paranigralis, the nucleus parabrachialis pigmentosus and the nucleus linearis caudalis. Subtype 7 (*Vglut2^+^Vip*^+^) is found in the periaqueductal grey and dorsal raphe nucleus. These detailed molecular characterizations of mDA neurons are especially useful when studying the selective vulnerability of certain subtypes in disease. For instance, *Sox6^+^Aldh1a1^+^* neurons were more prone to degeneration in a 1-methyl-4-phenyl-1,2,3,6-tetrahydropyridine (MPTP) mouse model of PD [46].

Similar to the rodent brain, the human mDA population is also highly heterogeneous. The first study profiling the human embryonic midbrain in 2016 found remarkable homology between the developing human and mouse midbrain [47]. However, there was a greater diversity of neuronal progenitors and radial glia in human embryos. Single-cell transcriptomics of the adult human midbrain affirms the presence of molecular subtypes, similar to those found in the mouse brain [47, 50]. A recent study carrying out single nuclei RNA sequencing and spatial transcriptomics of post-mortem human midbrain identified 10 molecular subtypes of mDA neurons [51]. Of note, the *CALB1^+^GEM^+^* cluster is likely a primate-specific subtype. Furthermore, the mDA subtypes affected in PD also correspond to those identified in PD rodent brains. One study found that *SOX6^+^ALDH1A1^+^* mDA neurons are more significantly reduced as compared to other mDA neurons [52]. More recently, another group utilized both single-cell transcriptomics and spatial transcriptomics to identify a subpopulation among the *SOX6^+^ALDH1A1^+^* mDA neurons that is more specifically associated with PD [51]. These neurons expressed angiotensin II receptor type 1a (AGTR1) and exhibited dysregulation in cellular stress pathways, suggesting a cell-intrinsic mechanism of PD susceptibility.

While these molecular subtypes broadly correspond to anatomical classifications, multiple subtypes may be found in the same brain region. The distinct localizations become significant as mDA neurons found in the SNc region are more selectively vulnerable to degeneration, as characteristically observed in PD pathology [53, 54]. Understanding the genetic heterogeneity of mDA neuron population can also facilitate the reprogramming of hPSCs into subtype-specific MLOs for more accurate disease modelling. To date, there are two published studies profiling the diversity of mDA neurons in MLOs at a single-cell resolution [38, 39]. Smits et al. demonstrated progressive dopaminergic neuronal maturation of MLOs over prolonged culture. They also found distinct neuronal populations apart from dopaminergic neurons, such as GABAergic and serotonergic neurons [38]. The neurons that developed in MLOs were also capable of spontaneous firing activity, suggesting maturation over time. More recently, Fiorenzano et al. produced a single-cell transcriptomic map of MLOs and were able to identify dopaminergic populations that correspond to the subtypes delineated in mouse and human midbrain atlases [39]. By carrying out a focal analysis on dopaminergic neurons in the MLOs, they identified graded expression of TH, dopamine transporter (DAT) and other mature mDA marker genes. mDA clusters that develop later in MLOs expressed more mature marker genes and ion channels necessary for synaptic function. Future studies may provide more in-depth characterizations of mDA neurogenesis and subtype-specific function in MLOs.

### Multi-lineage and multi-regional brain organoids

Although cerebral organoids are an improvement from 2D neuronal cultures, several important aspects are missing from this model. Neuronal function is influenced by interactions with other cell types, including astrocytes, oligodendrocytes, endothelial cells from the surrounding vasculature and microglia. During development, astrogenesis takes place after neurogenesis, and astrocytes require longer durations to mature in culture, with one study reporting astrocytic maturation at over 500 days *in vitro* [55]. Current protocols also lack the ability to generate mature oligodendrocytes in brain organoids [56]. While some protocols are able to produce glia cells that are transcriptomically similar to human samples [57], these glial populations develop only after long-term culture [58–61]. Hence, there is a need for methods to improve gliogenesis in brain organoids within experimentally tractable time frames. Given recent evidence that astrocytes and oligodendrocytes possess brain region-specific molecular and functional characteristics [62], updated protocols that can incorporate region-specific glia may improve the differentiation and survival of neurons in brain organoids.

Besides the slower development of glial cell types *in vitro*, cerebral organoids derived from hPSCs typically do not contain non-neural lineages. Thus, key cell types such as mesoderm-derived endothelial cells (ECs) that form the vasculature, and microglia, which are the brain’s resident immune cells are absent in most guided organoid-making protocols. The next generation of organoids will need to incorporate these elements and allow for modelling dynamic interactions between cell types and the surrounding microenvironment.

**Figure 2:**
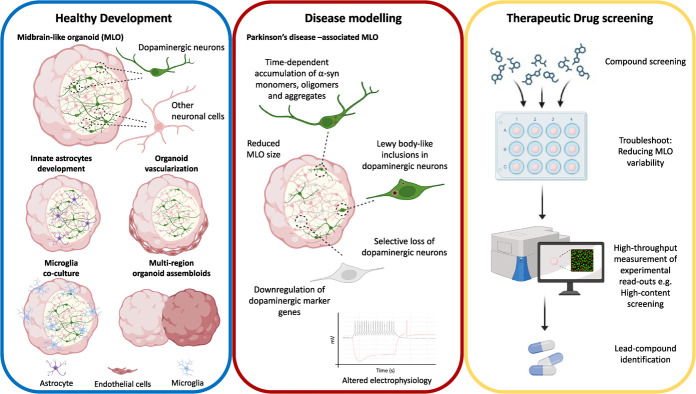
Applications of midbrain-like organoids (MLOs), created with BioRender.com. MLOs are increasingly used to model midbrain development and midbrain-associated diseases. To improve the biological relevance of MLOs, methods including accelerating astrocytes development, vascularization of MLOs, incorporation of microglia and the making of multi-regional brain organoids are being explored. Parkinson’s disease (PD) modelling using MLOs is also becoming increasingly common. Current PD MLOs models can mimic pathologies including selective dopaminergic neuronal loss, ⍺-synuclein accumulation, electrophysiology alterations and LB-like inclusions. Despite MLO technology being relatively new, it has shown promise as a tool for therapeutic screening purposes. Created with Biorender.com.

Guided differentiation protocols for generating brain organoids from hESCs produce reproducible and consistent organoids but also result in restricted regional identities. This limitation could be overcome by assembling organoids of different regions and is currently an active area of research. Assembloids of various combinations will allow scientists to model processes ranging from neural circuit assembly to neuro-immune interactions *in vitro*. In this section, we will be discussing recent advances in modelling neurovascular and neuroimmune interactions in brain organoids, as well as in generating multi-regional brain organoids (Fig. 2).

#### Vascularized organoids

Most brain organoids that have been generated, as reviewed in Section 1.2, lack neurovascular structures. Without a vascular system to deliver oxygen and nutrients, these organoids become structurally limited and inevitably develop a necrotic core as a result of long-term culture. Vasculature also regulates neurogenesis, and its presence has been associated with more neural progenitors [63–65]. Hence, in the absence of vascular structures in brain organoids, ECs do not survive for long and the proliferation and differentiation of neural progenitors could be impaired.

Several groups have independently devised ways to create vascularized organoids. Pham et al. [66] attempted a purely *in vitro* strategy by differentiating cerebral organoids and ECs from the same population of patient-derived iPSCs. The organoids and ECs were embedded together in matrigel, which provided structural support to the ECs and enabled their assembly into tubular structures and penetration into the organoid over time. A more recent attempt at generating vascularized organoids involved co-culturing human umbilical vein endothelial cells (HUVEC) and hPSCs to form mixed aggregates. The vascularized organoid subsequently generated was reported to have lower proportion of apoptotic cells [67]. Another group introduced a subset of *ETV2*-overexpressing hESCs directed towards the endothelial lineage, thus producing a mixed cerebral organoid with enhanced maturity containing both neural and endothelial-like cells [68].

In addition, transplanting brain organoids into animals could be a promising strategy to vascularize organoids. For instance, taking advantage of naturally occurring angiogenesis *in vivo,* Mansour et al. [69] grafted organoids into mouse brains and observed vascularization of the graft by host blood vessels. The grafted organoids also established synaptic connections with host neurons and displayed synchronized neuronal firing. Importantly, they also showed reduced apoptosis as compared to organoids cultured *in vitro*. Thus, the surrounding vasculature appears to promote organoid viability and functional maturation.

In another attempted by Sun et al. [70] to vascularize brain organoids was by establishing the fusion of vessel and brain organoids. In the resulting fusion organoid, CD31^+^ vessel-like structure was integrated within the brain organoid, leading to improved neurogenesis. This approach can also possibly be used to introduce functional microglia-like cells into brain organoids. At present, there are no MLO models incorporating vascular-like structures, but the introduction of vascular-like structures to MLOs will likely lead to improved cell survival and function. Nonetheless, there is no active perfusion even in organoids with vascular-like structures. Hence, other strategies to mitigate necrotic core formation in long-term organoid culture such as slicing [60, 71] or use of spinning bioreactors to improve oxygen delivery [72] can be considered.

#### Microglia integration

Microglia are brain-resident sentinel cells, essential for mediating a dynamic range of functions to maintain the central nervous systems (CNS). Accounting for 0.5% to 16.6% of the human brain cell population [73], microglial heterogeneity is evidenced both regionally and temporally by transcriptomic data [74]. Unlike other major cell types of the CNS (neurons, astrocytes, oligodendrocytes) that are derived from the neuroectoderm, microglia originate from erythro-myeloid precursors in the yolk-sac and populate the CNS during early embryogenesis [75]. Many studies have highlighted the importance of microglia–neuron communication in brain homeostasis; their impairment implicated in developmental (reviewed in [76]) and neurodegenerative disorders (reviewed in [77]). Several microglial models have been developed to recapitulate microglia–neuron interactions during brain development and pathologies, including *in vivo* mouse models [75, 78, 79], human iPSC (hiPSC)-based microglial mouse brain chimeras [80–82] and 2D neuron–microglia (microglial cell line or induced microglia-like cells [83–86]) co-cultures [87–90]. More recently, many groups have attempted to devise microglia-containing human brain organoids.

Due to the distinct origin of microglia, its presence is not recapitulated by guided organoid culture protocols, which mimic neuronal differentiation to produce region-specific neurons. As such, most of the microglia-containing human brain organoids are generated through the co-culturing of microglia with human brain organoids or the assembly of microglial progenitor cells with neural progenitor cells. However, Quadrato et al. reported that cerebral organoids generated from an unguided protocol—which was first described by Lancaster et al. [24] consist of a broad diversity of cells, including mesoderm-derived progenitors [91]. Subsequently, Ormel et al. [92] demonstrated that cells of microglial identity can innately develop within the cerebral organoids following minor modifications of the unguided protocol. Nonetheless, there is currently scarce details on midbrain-specific microglia-containing human brain organoids. To our knowledge, the only such protocol was described by Sabate-Soler et al. [93], who co-cultured hiPSC-derived macrophage progenitors with human midbrain organoid at 15 days post DA differentiation. Promisingly, the incorporation of microglia into the midbrain organoid led to improved neuronal maturation and functionality, presenting an improved model for studying neuroinflammation-associated conditions of the midbrain (e.g. PD).

#### Multi-region organoids

Apart from supporting cellular interactions between neural and non-neural cells, current organoid models also need to capture regional crosstalk between brain regions. Fusing multiple region-specific brain organoids to form assembloids may allow us to observe cellular migration and neural circuit formation. Several groups have pioneered the assembly of region-specific brain organoids to form multi-region assembloids. These approaches are modular and leverage on the self-organizing properties of cells in 3D culture. To form assembloids, regionally patterned organoids are cultured separately before allowing them to fuse and form long-range cellular interactions. Neurons within assembloids also demonstrate more mature electrophysiological properties [94].

Most of the literature on assembloids involves cortical organoids. The earliest assembloid was a fusion of dorsal and ventral forebrain organoids by two research groups [95, 96]. The cortex contains two main neuronal populations: excitatory glutamatergic pyramidal neurons and inhibitory GABAergic interneurons. The excitatory neurons originate from the progenitors in the dorsal forebrain, whilst the inhibitory interneuron population develops from the ventral forebrain progenitors. During development, interneurons undergo long-range migration from their ventral location to innervate the dorsal cortex. With a dorsal-ventral forebrain assembloid, the migration and integration of the interneurons into dorsal cortical circuits could be visualized. Using live imaging, both groups observed unidirectional migration of GABAergic neurons from ventral forebrain organoids into the dorsal region [95, 96]. Of note, this migration pattern was less apparent in 2D co-cultures of the two neuronal populations, suggesting that 3D cultures better simulate *in vivo*-like neuronal interactions. Hence assembloids represent a simplified model for uncovering the molecular mechanisms underlying neural circuitry.

Several other types of assembloids have been generated since then. The first human cortico-striatal assembloid was reported in 2020 [94], in which cortical neurons projected into the striatal region, recapitulating *in vivo* projection patterns. Cortical organoids were also fused with spinal cord organoids to form cortico-spinal assembloids [97] wherein cortical neurons similarly projected into the spinal cord region. A complete neuromuscular unit was formed by creating an assembloid of cortical organoids, spinal cord organoids and skeletal muscle spheroids. Spontaneous skeletal muscle contractions were recorded from the assembloids and the activity was more coordinated in the corticomotor organoid compared to skeletal muscle spheroids alone. These studies demonstrate that multi-region assembloids can exhibit features of the human brain and enhance the functional maturity of cultured cells.

Strikingly, the midbrain is absent in the assembloid studies described here. Regional assembloids to date have not involved modelling midbrain connectivity with other regions. The midbrain projects to multiple brain areas. Earlier work uncovered the projections and inputs of midbrain neurons with anatomical tracing and molecular labelling tools [98–100]. It is involved in two pathways that regulate motivation, reward and addiction: namely the mesocortical pathway from the midbrain to the cortex [101, 102] and the mesolimbic pathway from the midbrain to limbic structures such as the ventral striatum and amygdala [103, 104] The midbrain is also involved in the nigrostriatal pathway (midbrain to dorsal striatum), a well-known motor circuit that degenerates in PD [105, 106]. However, in primates these mesencephalic pathways are increased in complexity and size, as dopaminergic neurons from both SNc and VTA contribute to innervating the cortex, striatal and limbic structures. As regional assembloids utilize a modular approach, combining MLOs with striatal organoids can be a viable alternative to animal models to study the molecular mechanisms of midbrain development and degeneration.

## PATHOLOGICAL RELEVANCE OF MLOS

Besides allowing the recapitulation of complex physiology of the human midbrain, midbrain-like organoids also present as a promising strategy for modelling midbrain-associated disorders. PD is the second most common neurodegenerative disorder characterized by neuropathologies including selective loss of mDA neurons in the SNc, the presence of Lewy bodies (LBs) and intracellular inclusions of misfolded alpha-synuclein (α-syn) aggregates [107]. Due to reduced dopamine levels resulting from mDA cell death, patients present with classic motor symptoms including resting tremors, muscle stiffness and bradykinesia [3]. In most cases, PD develops as an idiopathic disease; inheritable genetic causes of PD can be observed in only 10–15% of all patients [108]. To date, familial-linked cases of PD have been associated with rare genetic variants of over 20 genes [109], of which, mutations in *SNCA*, *LRRK2, GBA, VPS35, DJ-1, PARKN* and *PINK1* have most commonly been implicated in disease-associated pathways (reviewed in [110]). However, the molecular mechanisms underlying the course of disease remain elusive. Therefore, generating genetically modified and patient-derived MLOs constitute a valuable tool for unveiling mechanisms underlying PD-linked pathogenesis (Fig. 2).

### Parkinson’s disease genetic models

Since the protocol for generating hPSC-derived mDA neurons was established [111], many studies have attempted to characterize PD-associated phenotype and molecular changes using 2D culture of patient iPSC-derived mDA neurons (reviewed in [112, 113]). These studies significantly advanced our understanding of disease-associated mechanisms and contributed to the identification of therapeutic targets. However, the inability of monolayer cultures to self-organize and challenges in maintaining long-term 2D cultures limit the relevance of 2D neuronal culture as a model of the highly complex and structurally organized human brain.

In recent years, the pervasiveness of brain organoids has led to increased use of MLOs in PD modelling. Predominantly, studies have characterized MLOs generated from hPSCs carrying PD-associated mutations such as *LRRK2* [43, 114, 115], *PRKN* [116]*, SNCA* [117–119]*, GBA1* [118]*, DNAJC6* [120] and *PINK1* [121]. The earliest proof of concept studies demonstrated that MLO models of PD can recapitulate different aspects of disease-relevant pathologies. *SNCA* triplication MLOs is shown to recapitulate PD-associated α-syn pathologies. Mohamed et al. [119] demonstrated that patient-derived *SNCA* triplication MLOs exhibit time-dependent accumulation and aggregation of α-syn, including oligomeric α-syn and α-syn phosphorylated at serine 129 (pS129Syn). Notably, the increase in neuronal and glial pS129Syn aggregates is accompanied by a selective loss in the number of TH^+^ DA neurons [119]. In parallel, similar observations were also made in our study; we showed that *SNCA* overexpression in MLOs result in the selective degeneration of FOXA2^+^/TH^+^ mDA neurons. In addition, we observed an increased proportion of TH^+^ mDA neurons with α-syn inclusions and increased levels of insoluble α-syn [118]. CRISPR-engineered *GBA1*^−/−^ MLOs also exhibited similar properties. We also attributed the reduction in TH^+^ mDA neurons to increased apoptosis, indicated by increased expression of apoptotic markers. Intriguingly, when *GBA*^−/−^ mutation was combined with *SNCA* overexpression, the resulting MLOs produced α-syn-containing LB-like inclusions, similar to those seen in PD, in TH^+^ mDA neurons [118]. The finding was further validated on patient-derived MLOs. Patient-derived *SNCA* triplication MLOs treated with conduritol-b-epoxide to inhibit GCase activity and patient-derived *GBA1*-N370S MLOs induced to overexpress wild-type α-syn both exhibited LB-like inclusion morphology [118]. More recently, a study by Becerra-Calixto et al. [117] suggested that the long-term culturing of PD MLOs harbouring *SNCA* triplication alone was potentially sufficient for the recapitulation of LB-like morphology. The authors noted that patient-derived *SNCA* triplication MLOs manifest pS129^+^/αSyn^+^ spherical structures upon culturing for 180 days; these spherical structures have smooth edges juxtaposed to the nucleus which resemble LB morphology observed in PD patients’ brain sections.

Microarray analysis of CRISPR-engineered *LRRK2*-G2019S MLOs revealed a transcriptional signature of LRRK2-associated sporadic PD patient-derived brain tissue [43]. Phenotypically, the MLOs also exhibited aberrant localization of pS129Syn, decreased neurite lengths and reduced mDA neuron-specific markers including *TH, AADC, VMAT2* and *DAT* [43]. Concurrently, Smits et al. observed significantly reduced DA network complexity among TH-positive neurons in patient-derived *LRRK2*-G2019S MLOs [114], a phenomenon also observed in PD patients’ brains [122]. Interestingly, Smits and colleagues found an increase in FOXA2-positive progenitors in *LRRK2*-G2019S MLOs [114] which they hypothesized to be a compensatory mechanism [123] for impaired mDA neuron specification resulting from *LRRK2* mutation. In another study, single-cell RNA sequencing analysis of CRISPR-engineered *LRRK2*-G2019S MLOs revealed an accelerated differentiation phenotype accompanied with impaired maturation of DA neurons, as seen by reduced *TH* expression [115]. *LRRK2*-G2019S MLOs also deviate from the embryonic pseudotime development trajectory while wild-type MLOs trial closer along the development trajectory [115]. This could potentially reflect a neurodevelopmental element [124] of PD which influences disease susceptibility.

Loss-of-function mutations of *DNAJC6* have also been demonstrated in MLOs to reflect neurodevelopmental defects leading to PD-like phenotypes. Transcriptomic analyses of CRISPR-engineered *DNAJC6* MLOs showed the specific downregulation of mDA neurons developmental genes [120]. Further analyses revealed an early WNT-LMX1A impairment, contributing to the generation of vulnerable mDA neurons as a result of reduced dopaminergic identity. Additionally, multielectrode array (MEA) recording on neurons in *DNAJC6* MLOs showed increased firing frequencies [120], resembling neuronal characteristics observed in progressive PD [125]. Loss of DNAJC6 also contributes to autolysosomal defect-associated aberrations in α-syn degradation, leading to the manifestation of α-syn pathology [120]. Functionally, patient-derived *PINK1* mutant MLOs exhibited reduced mDA neuron maturation; whole cell patching measured reduced neuronal action potential numbers and amplitude relative to wild-type MLOs [121].

Separately, MLOs can also recapitulate non-neuronal pathologies of PD. A study by Kano et al. showed that patient-derived *PRKN* MLOs contain reduced number of GFAP^+^ astrocytes, mimicking the observation made in the substantia nigra of patients with *PRKN* mutation [116]. Besides MLO models of known PD mutations, a study by Chlebanowska et al. [126] characterized MLOs derived from patients with idiopathic PD to demonstrate the influence of idiopathic background PD on MLOs generation. Strikingly, within 39 days in culture, patient-derived MLOs express *TH* at levels 3.6-fold lower than healthy volunteer derived MLOs. The difference in *TH* expression increased to 5-fold on day 49 in culture. The expression levels of genes regulating mDA neuron development, including *FOXA2, LMX1A and PTX3,* in patient-derived MLOs also showed significant differences from healthy control during the differentiation process [126]. These intrinsic gene expression changes observed in patient-derived MLOs could offer clues to understanding their vulnerability to PD.

In the span of about 5 years, many MLO models of PD have been derived, each recapitulating various pathological aspects of PD. Over the years, protocols have also been revised to better model MLOs with PD. Starting with early studies that demonstrate the selective loss of mDA neurons and aberrant accumulation of α-syn in MLOs, advanced, new generation of MLOs are being adopted, that enables more accurate recapitulation of PD pathologies such as LB deposits. The capability to model the hallmark features of PD will provide a more comprehensive understanding of disease mechanisms and better model for the identification of therapeutic targets and interventions.

## THERAPEUTIC RELEVANCE OF MLOS

There are currently no available disease modifying interventions for the amelioration or reversal of PD. The drugs approved for use on PD patients largely provide symptomatic relief by increasing dopamine levels, with the mode of action ranging from dopamine precursors, decarboxylase inhibitors, catechol-o-methyl-transferase inhibitors, dopamine agonists, monoamine oxidase-B inhibitors, N-methyl-D-aspartate antagonists, adenosine A2A antagonists and anticholinergics (reviewed in [127]). Nonetheless, levodopa (L-DOPA), FDA approved since 1975, remains the most effective drug for addressing motor symptoms that arise in PD [128]. With an ever-increasing PD incidence rate, there is a pressing need to identify better drug targets that can alter the course of disease. However, complex, multi-faceted pathological processes that extend over prolonged periods in PD pose significant challenges in the identification of underlying mechanisms and development of effective therapeutic strategies. Moreover, poor translatability from preclinical models to human patients, partly due to the inadequacy of *in vitro* and disease animal models [129], can confound the validation of drug target effects. In this section, we will discuss the potential of MLOs as an improved model for selecting lead drug candidates for PD treatment (Fig. 2).

### MLOs as a tool for drug screening

For a long time, animal models have been viewed as a tool to learn about physiological processes and bridge the gap between basic and clinical research. Historically, animal studies were required as part of the drug approval process. This has since been ruled against [130] potentially due to its outdated underlying scientific principles [131]. While animal models clearly benefit the study of disorders that occur naturally in both humans and animals, the validity of animal models in complex diseases of humans is largely controversial [132]. Specific to PD alone, over 20 mouse models have been generated to mimic the disorder. Across these models, the animals present enormous variability in their recapitulation of pathological phenotype as well as the order of onset and progress of pathological features [133]. Such variability eventually poses significant challenge for the convergence of findings obtained from across different animal models of disease. Thus, there is a need for improved preclinical models that consistently recapitulate both pathological features and the underlying mechanisms of PD.

While the underlying mechanism of PD remains to be fully elucidated, the development of human cell reprogramming technology in 2007 [134] has ushered in a new wave of studies involving hiPSC-derived models of disease. Since then, various hiPSC models recapitulating PD phenotype were generated to study disease pathogenesis. Subsequently, many drug discovery studies have involved rescuing PD-associated phenotype in hiPSC-derived models with selected compounds or small molecules. These studies lay the foundation for the use of hiPSC-based models in future drug-screening projects. More recently, the refinement of MLO generation protocols (refer to Section 1) has further improved the biological relevance (refer to Section 2) of hiPSC-based models for the investigation of midbrain biology. Several MLOs that recapitulate key pathological features of PD, including the selective loss of DA neurons and the presence of α-syn pathology (refer to Section 3), have also been generated. These pathological manifestations in MLOs present measurable benchmarking read-outs for assessing the effects of drug candidates on PD-associated phenotype.

Even though the MLO technology is still relatively new, a few studies in recent years have already demonstrated the potential use of MLOs as a tool for PD drug discovery. In a publication by Jarazo et al. [135], proteomics analysis of PD patient-derived *PINK1* MLOs to investigate dysregulation in pathways identified reduced autophagy and mitophagy capacity as potential disease mechanisms. Subsequently, treating *PINK1* MLOs with 2-hydroxypropyl-β-cyclodextrin (HP-β-CD), a repurposed compound previously reported to modulate autophagy, rescued TH^+^ DA neurons proportion in MLOs [135]. Furthermore, treating toxicity-induced PD mice model with HP-β-CD also conferred benefits reflected by reduced loss of TH^+^ neurons [135]. In a separate study, vesicular storage dysfunction was identified as a potential disease mechanism using young-onset PD (YOPD) patient-derived MLOs [121]. Treatment with amantadine, a known PD drug, ameliorated this dysfunction [121]. Subsequently, also identified phorbol 12-myristate 13-acetate as a promising therapeutic compound for modulating vesicular activities [121]. These studies demonstrate the emergence of PD MLOs as a prospective tool for discovering disease mechanisms and identifying targeted therapeutics.

While the current findings are promising, we need to recognize the limitations of these models and refine them further. It is known that environmental and other external factors can introduce batch variations among cultured brain organoids, impacting the accuracy of drug screening studies (reviewed in [136]). Therefore, simplifying or improving brain organoid generation methods could potentially solve the problem. For example, Ha et al. generated structurally simple but homogenous midbrain-like simplified brain organoids (simBOs) using hPSC-derived primitive neural stem cells (pNSCs) [137]. By 2 weeks of differentiation, TH^+^ and MAP2^+^ DA neurons were shown to be distributed throughout the midbrain-like simBOs. To model PD, Ha et al. [137] further generated midbrain-like simplified brain organoids (simBOs) from pNSCs derived from patient-derived *LRRK2*-G2019S iPSCs which exhibited a specific reduction in DA neurons and increased LRRK2 activity. Treatment with PFE-360 rescued the reduced dopamine release in the PD-simBO [137]. Taken together, this study demonstrates that simplifying protocols to generate more homogenous brain organoids could reduce experimental variability and improve translatability. New techniques could also be adopted to improve detection accuracy of disease phenotypes in MLOs. For example, Zanetti et al. [138] developed a novel electrochemical sensing method for the sensitive detection and monitoring of small changes in dopamine release between healthy and *LRRK2*-PD brain organoids with or without pharmacological intervention. Such innovations alongside the development of MLO technology will help to facilitate more accurate and effective use of MLOs for drug screening purpose.

Drug screening can be a long and tedious process. Several studies have adopted high-throughput technologies to scale-up the drug screening process with 3D brain organoids. In the approach described by Durens et al. [139], high-content imaging (HCI) was adopted to assess the cellular compositions and connectivity, and neurite length and branching of cortical organoids. Their method involved transferring of developing organoids into polytetrafluoroethylene (PTFE) cell-culture inserts which causes thinning of the organoids thus allowing increased light penetration and reduced refraction. In addition, HCI can also be multiplexed with multi-electrode arrays (MEAs) and single-cell calcium imaging to monitor network and individual cellular activity respectively [139]. In another publication, Renner et al. [140] presented a fully automated workflow for pharmacological screening in MLOs. The workflow automation begins with the generation of MLOs using a robotic automated liquid handling system which gives rise to MLOs with minimal intra and inter-batch variability in MLO size, morphology, cell composition and cell organization. The homogeneity of the MLOs generated was further reflected during the functional analysis of MLOs by calcium imaging, multielectrode array and voltage patch-clamping which demonstrated reproducible calcium and electrical activities [140]. Furthermore, the MLOs maintained through automated handling can be subjected to an automated process of whole-mount immunostaining and tissue clearing for optical HCI and quantification [140]. These phenotypic screening platforms demonstrate ways to overcome labour-intensive and time-consuming methods such as organoid sectioning for MLO-based drug screening experiments in the future.

## FUTURE OF MIDBRAIN ORGANOIDS

3D midbrain organoids are gaining traction as a powerful tool to understand human midbrain development, maturation and disease-associated degeneration. Notwithstanding the encouraging insights this technology has already delivered, the current generation of MLOs have several limitations, which we enumerate in the following paragraphs. Future strategies addressing these issues, where known, are provided as well.

**Biological relevance**: Current evidence points to an overall similarity between mDA neurons in MLOs and their *in vivo* counterparts but also reveals significant differences. Of note, a substantial gap exists in the functional maturity of the mDA neurons in MLOs. *In vivo*, mDA neurons are known to exhibit specialized electrophysiological properties, such as prominent sag currents, rebound hyperpolarization and the ability to release dopamine (DA) tonically or in bursts [141, 142]. These functionally mature traits of *in vivo* mDA neurons are largely absent in cultured MLOs, although it is possible to obtain them under prolonged culture conditions. Nonetheless, it is possible that prolonged culture may result in more functionally mature MLOs. In fact, several groups have reported success in maintaining viable and functional brain organoids for more than 1 year [143, 144]. However, whether the long-term culturing of MLOs can replicate *in vivo*-like electrophysiological properties remains to be demonstrated. Future efforts aimed at producing the full functional spectrum of mDA neurons are clearly warranted.

Surprisingly, NM, a dark pigment which gradually accumulates in humans from childhood, can be found in MLOs after just four months of culture [35]. Given that organoids are under ‘stress’ [145], it is tempting to assume that NM are induced by stress but this remains to be experimentally confirmed. Also, MLOs lack the intricate interaction between neurons and other cell types or need to interact with other brain structures to become more functionally mature. As mentioned before, other resident midbrain cell types, including astrocytes, oligodendrocytes, ECs and microglia need to be enriched in MLOs. Among them, oligodendrocytes have not been reported in any MLO systems, but sequencing studies have suggested these cells constitute a significant proportion of the human midbrain [53, 54]. Intriguingly, earlier work indicate that axonal projections of mDA neurons are unmyelinated [146, 147]. Future work should generate oligodendrocytes within MLOs and investigate their effects on mDA neurons.

Lastly, mDA axons innervate and arborize extensively in the striatum [148], forming the nigrostriatal pathway. This anatomical feature poses a huge energy demand on mDA neurons, forming the basis of metabolic vulnerability of mDA neurons in PD [149]. However, mDA neurons in MLOs do not produce such elaborated arborization and lack the appropriate striatal cells to project to. Fusion midbrain and striatal organoids are being studied to reconstruct elements of the nigrostriatal pathway *in vitro*.

**Pathological relevance**: MLOs are more similar to the human brain, in both molecular and structural features, as compared to 2D neuronal cultures. Thus, they have greater potential to reveal the molecular mechanisms and cellular pathways underlying PD. As discussed in Section 3, mutant MLOs could even develop LB-like inclusions, closely reflecting patient pathologies. Although these MLO models of disease do not precisely mimic physiological conditions, they can represent accelerated disease progression and be used to dissect various aspects of PD, such as the genetic interactions of risk genes and the ontogeny of LB. Future studies may employ more of mutant-harbouring MLOs, results from which may be integrated into convergent modules and used to develop therapies.

The similarity of MLOs to the human fetal brain poses a challenge when using them to model adult-onset diseases like PD. Hence, it is crucial to account for age-related characteristics when creating *in vitro* organoid models of disease. Be it by progerin-induced ageing [150] or silencing of telomerase activity [151], accelerating the ageing of MLOs in culture will be desired. Transplanting MLOs into animal brains, as recently demonstrated in the case of cortical neurons [152–154] and organoids [155–160], also offers an attractive means to enhance neuronal maturity as well as functional connectivity. A group comparing the transplantation of dissociated cells and intact organoids found that organoid grafts more robustly integrated with host vascularization, which aided their survival *in vivo* [155]. This ‘hybrid’ model would be a significant asset of the field if it indeed is shown to reproduce human pathology more faithfully.

**Therapeutic relevance:** As the number of studies engaging brain organoids for therapeutics continue to increase, work on the generation of MLO models is likely to experience a similar upward trajectory. However, as discussed previously, large-scale compound screening is currently unrealistic until new generation of MLOs with improved homogeneity and robust readouts become available. We foresee that novel bioengineering advances (such as microfluidics) as well as innovative molecular probes (such as genetically encoding DA sensor), will aid greatly in this [161, 162]. Separately, applying genome-editing technology, such as CRISPR, to MLOs may lead to the identification of novel targets that may modify disease pathology. Furthermore, MLOs could be used to study gene–environment interactions, at the very least in terms of neurotoxins, potentially yielding novel therapeutic avenues. Finally, MLOs contain many other cell types, such as VTA-like mDA neurons, which are central to neural circuitry underlying reward, addiction and depression [163, 164]. As the cellular composition of MLOs becomes clearer and more diversified, they are more commonly being used to model diseases beyond Parkinsonism.

Another area MLOs may be useful is for cell replacement therapy in PD. So far reasonably encouraging outcomes have been achieved with dissociated 2D mDA progenitor [165]. There have been continued efforts to optimize various aspects of the transplantation process to restore nigrostriatal circuitry in animal models of PD. One study demonstrated that hESC-derived mDA neuronal grafts showed comparable efficacy to fetal-derived ventral midbrain grafts in terms of restoring motor function in 6-hydroxydopamine (OHDA) lesioned adult rats [166]. Another group demonstrated similarly promising transplantation results with hiPSC-derived mDA cells [17]. These early works provided strong support for the use of hPSC-derived dopaminergic neurons in transplantation models of PD. Since then, there have been numerous attempts at transplanting hPSC-derived dopaminergic grafts [167–169], including some that transplanted grafts into non-human primates [170–172]. To progress towards human clinical trials, factors related to both grafted cells and the host brain environment have to be optimized [173–175]. Transplanted cerebral organoids have also proven to be effective in producing phenotypic changes, as in the case of motor function rescue of traumatic brain injury (TBI) rat models [159], and vision in visual cortex-lesioned mice [176]. MLOs thus hold the same potential in improving outcomes for PD animal models, and eventually human PD trials of cell replacement therapy.

With FDA abolishing the long-held requirement of animal testing for drug development, it is a particularly exciting era for organoid technology as it holds great potential to become a full-fledged preclinical model prior to human trials. Since MLOs can be derived from individual patients using iPSC technology, they can serve as a platform for precision medicine — from identifying drug treatments that will be effective against an individual’s genetic and epigenetic background to potential cell therapy. Although current MLO models are insufficient to recapitulate the real midbrain tissue, understanding and discussing the limitations of organoid technology will undebatably motivate breakthroughs in the next generation of MLOs in the near future.

## Supplementary Material

Web_Material_kvad009
