# Supplementary material for: Midbrain organoids—development and applications in Parkinson’s disease
Source: Oxf Open Neurosci. 2023 Aug 18;2:kvad009. doi: 10.1093/oons/kvad009 (PMC10913847; doi:10.1093/oons/kvad009)
Supplement: Web_Material_kvad009 [file Web_Material_kvad009.docx]

**REVIEWER COMMENTS**

**OXFNSC-2023-006.R2- Midbrain organoids – Development and Applications in Parkinson’s Disease**

**Original submission - OXFNSC-2023-006**

**Reviewer 1**

Comments to the Author

In is review, the authors introduced the developing of midbrain-like organoids (MLOs) models, summarized their biological relevance and applications in disease modeling and therapies. Additionally, they discussed the limitations and potential improvements of MLOs technology in the future. Overall, authors do a commendable job in summarizing the state-of-art in the field with considerable details on the protocols and findings. I only have a few comments for the authors to improve their manuscript:

Major point

1. In Figure1, the timeline of unguided v.s. guided organoid protocol is not correct. The protocols developed by Kadoshima et. al., 2013 and Pasca et. al., 2015 used SMAD inhibitor and some growth factors to restrict cell fates and should be considered as guided protocols. Qian et al. developed guided protocol for several region specific brain organoids. It should also include subregions specific organoids, such as Huang et al. 2022 for arcuate nucleus organoid and Kiral et al. 2023 for ventralized thalamic organoids.

2. The structure of section 1- Generating Human Dopaminergic Neuronal Models is a little confusion. Clearly, section 1.1- Generating Three-dimensional Brain Organoids does not belong to the topic of section 1. Instead, it should be part or introduction of section 1.2- Development of Midbrain-like Organoids. I suggest that the authors reorganize section 1 by firstly introducing 2D dopaminergic neuronal models and then 3D Midbrain-like Organoids.

Minor points

1. A typo in Page 2 Line 38 “tand” should be “and”

2. Page 3 line 31, the authors may cite the protocol developed by Vannary Tieng (https://doi.org/10.1089/scd.2013.0442) as well. Besides, Matrigel embedding is not a common feature of these protocols since several of them (for example: L. M. Smits et al.; X. Qian et al.; Tieng et al.) didn’t use it. It’d be better to remove the sentence “Matrigel is added to the culture ……” in line 35-36.

3. Page 5 line 48-49, the statement that “cerebral organoids derived from hPSCs typically do not contain non-neural lineages” is not true for unguided protocol that can generate microglia, which has also been discussed in this manuscript in section 2.2.2.

4. Page 10 line 41-42, delete the repeated sentence “were significantly different in patient derived MLOs.”

5. Page 15 line 23-24, they mention transplanted organoids as a method to rescue motor and visual function in the review but do not give any references.

**Reviewer 2**

Comments to the Author

 Review of:

“Midbrain organoids and disease”

In this review, the author(s) summarize the current literature on 2D and 3D models to study midbrain dopaminergic neuronal differentiation and their use in studying neurological disorders such as Parkinson’s disease (PD).

This is a useful review with a detailed and very well-written section 3 summarizing the salient features of various studies using 2D/3D neuronal and organoid models to study PD in vitro. The therapeutic relevance and future scope of midbrain organoids is also well written but Sections 1 and 2 need refinement as detailed below.

Major comments

1. The review title is too generic. To better reflect the authors' focus on the various 2D/3D models of mid-brain and dopaminergic neuronal development and their relevance in studying PD, the title should be revised to incorporate this aspect.

2. The authors have summarized the existing methodologies but the review should include more authors’ own opinions on how newer and more robust protocols can be developed based on existing understanding of midbrain and dopaminergic neuronal development as there are not many studies in this area.

Below are summarised various instances in Sections 1 and 2 where the authors should elaborate more:

a. Section 1 and Section 1.2 - The authors summarise the various protocols but do not discuss how the existing 2D/3D protocols mimic neurodevelopment in a detailed manner. A detailed molecular analysis will help identify existing lacunae in the protocols and help pave the way for designing future additions to the existing protocols. Suggestion: A schematic if added to this section will vastly improve the import of the section.

b. P3, Line 53- what are these midbrain patterning factors? Discussion on their role would be useful.

c. P4, Line 11- The title is too generic /broad. This should be amended to be more specific.

d. P4, Line 13. The entire section is on the various neuronal subtypes in the mouse and human midbrain and not MLOs. This should be amended.

e. P5, Lines 21-28 - The authors have summarised the results of Smits et al., and Fiorenzano et. al in a few lines but since the major focus of the review is the development of MLOs and their usage in studying disorders, the review should include a detailed molecular analysis and discussion on how the two studies demonstrate the ability of MLOs to mimic dopaminergic neurogenesis.

f. Section 2.2.3 – Multi-region organoids- a major portion of this section discusses muti-region organoids in the dorsal forebrain. The authors notice the absence of studies modelling mid-brain connectivity but the lines from 30-40 lack references. The authors should discuss here specific animal studies showing how this connectivity is important for overall brain function and also how this pathway is degenerated in PD to bolster the need for MLOs with striatal organoids to gain a human development and disease perspective.

g. P9 Line 21- Insert appropriate references. The citation of the paper should be the place at which the evidence is first described, not at the last.

Minor comments

1. P2, Line 35- remove Development of Midbrain-like Organoids

2. P2, Line 38- Correct “tand” to “and”

3. P11- Line 15- Correct “tcomplex” to “complex”

**Decision letter - OXFNSC-2023-006**

20-Jun-2023

Dear Dr. Sun,

Manuscript ID OXFNSC-2023-006 entitled "Midbrain organoids & disease" which you submitted to the Oxford Open Neuroscience, has been reviewed. The comments of the reviewer(s) are included at the bottom of this letter.

The reviewer(s) have recommended publication, but also suggest some minor revisions to your manuscript. Therefore, I invite you to respond to the reviewer(s)' comments and revise your manuscript.

Please note that this journal operates with transparent peer review. This means that if your submission is accepted for publication, the full peer review history of your article will publish online alongside your article. This includes reviewer comments, editor decision letters, and your author responses.

To revise your manuscript, log into https://mc.manuscriptcentral.com/oxfnsc and enter your Author Centre, where you will find your manuscript title listed under "Manuscripts with Decisions." Under "Actions," click on "Create a Revision." Your manuscript number has been appended to denote a revision.

You may also click the below link to start the revision process (or continue the process if you have already started your revision) for your manuscript. If you use the below link you will not be required to login to ScholarOne Manuscripts.

*** PLEASE NOTE: This is a two-step process. After clicking on the link, you will be directed to a webpage to confirm. ***

https://mc.manuscriptcentral.com/oxfnsc?URL_MASK=170cb08e8e624813b6fcc9ad67034c34

You will be unable to make your revisions on the originally submitted version of the manuscript. Instead, revise your manuscript using a word processing program and save it on your computer. Please also highlight the changes to your manuscript within the document by using the track changes mode in MS Word or by using bold or colored text.

Once the revised manuscript is prepared, you can upload it and submit it through your Author Centre.

When submitting your revised manuscript, you will be able to respond to the comments made by the reviewer(s) in the space provided. You can use this space to document any changes you make to the original manuscript. In order to expedite the processing of the revised manuscript, please be as specific as possible in your response to the reviewer(s).

IMPORTANT: Your original files are available to you when you upload your revised manuscript. Please delete any redundant files before completing the submission.

Because we are trying to facilitate timely publication of manuscripts submitted to the Oxford Open Neuroscience, your revised manuscript should be uploaded as soon as possible. If it is not possible for you to submit your revision in a reasonable amount of time, we may have to consider your paper as a new submission.

Once again, thank you for submitting your manuscript to the Oxford Open Neuroscience and I look forward to receiving your revision.

Sincerely,

Dr. Orly Reiner

Senior Editor, Oxford Open Neuroscience

orly.reiner@weizmann.ac.il, orly.reiner@weizmann.ac.il

Reviewer: 1

Comments to the Author

In is review, the authors introduced the developing of midbrain-like organoids (MLOs) models, summarized their biological relevance and applications in disease modeling and therapies. Additionally, they discussed the limitations and potential improvements of MLOs technology in the future. Overall, authors do a commendable job in summarizing the state-of-art in the field with considerable details on the protocols and findings. I only have a few comments for the authors to improve their manuscript:

Major point

1. In Figure1, the timeline of unguided v.s. guided organoid protocol is not correct. The protocols developed by Kadoshima et. al., 2013 and Pasca et. al., 2015 used SMAD inhibitor and some growth factors to restrict cell fates and should be considered as guided protocols. Qian et al. developed guided protocol for several region specific brain organoids. It should also include subregions specific organoids, such as Huang et al. 2022 for arcuate nucleus organoid and Kiral et al. 2023 for ventralized thalamic organoids.

2. The structure of section 1- Generating Human Dopaminergic Neuronal Models is a little confusion. Clearly, section 1.1- Generating Three-dimensional Brain Organoids does not belong to the topic of section 1. Instead, it should be part or introduction of section 1.2- Development of Midbrain-like Organoids. I suggest that the authors reorganize section 1 by firstly introducing 2D dopaminergic neuronal models and then 3D Midbrain-like Organoids.

Minor points

1. A typo in Page 2 Line 38 “tand” should be “and”

2. Page 3 line 31, the authors may cite the protocol developed by Vannary Tieng (https://doi.org/10.1089/scd.2013.0442) as well. Besides, Matrigel embedding is not a common feature of these protocols since several of them (for example: L. M. Smits et al.; X. Qian et al.; Tieng et al.) didn’t use it. It’d be better to remove the sentence “Matrigel is added to the culture ……” in line 35-36.

3. Page 5 line 48-49, the statement that “cerebral organoids derived from hPSCs typically do not contain non-neural lineages” is not true for unguided protocol that can generate microglia, which has also been discussed in this manuscript in section 2.2.2.

4. Page 10 line 41-42, delete the repeated sentence “were significantly different in patient derived MLOs.”

5. Page 15 line 23-24, they mention transplanted organoids as a method to rescue motor and visual function in the review but do not give any references.

Reviewer: 2

Comments to the Author

Review of:

“Midbrain organoids and disease”

In this review, the author(s) summarize the current literature on 2D and 3D models to study midbrain dopaminergic neuronal differentiation and their use in studying neurological disorders such as Parkinson’s disease (PD).

This is a useful review with a detailed and very well-written section 3 summarizing the salient features of various studies using 2D/3D neuronal and organoid models to study PD in vitro. The therapeutic relevance and future scope of midbrain organoids is also well written but Sections 1 and 2 need refinement as detailed below.

Major comments

1. The review title is too generic. To better reflect the authors' focus on the various 2D/3D models of mid-brain and dopaminergic neuronal development and their relevance in studying PD, the title should be revised to incorporate this aspect.

2. The authors have summarized the existing methodologies but the review should include more authors’ own opinions on how newer and more robust protocols can be developed based on existing understanding of midbrain and dopaminergic neuronal development as there are not many studies in this area.

Below are summarised various instances in Sections 1 and 2 where the authors should elaborate more:

a. Section 1 and Section 1.2 - The authors summarise the various protocols but do not discuss how the existing 2D/3D protocols mimic neurodevelopment in a detailed manner. A detailed molecular analysis will help identify existing lacunae in the protocols and help pave the way for designing future additions to the existing protocols. Suggestion: A schematic if added to this section will vastly improve the import of the section.

b. P3, Line 53- what are these midbrain patterning factors? Discussion on their role would be useful.

c. P4, Line 11- The title is too generic /broad. This should be amended to be more specific.

d. P4, Line 13. The entire section is on the various neuronal subtypes in the mouse and human midbrain and not MLOs. This should be amended.

e. P5, Lines 21-28 - The authors have summarised the results of Smits et al., and Fiorenzano et. al in a few lines but since the major focus of the review is the development of MLOs and their usage in studying disorders, the review should include a detailed molecular analysis and discussion on how the two studies demonstrate the ability of MLOs to mimic dopaminergic neurogenesis.

f. Section 2.2.3 – Multi-region organoids- a major portion of this section discusses muti-region organoids in the dorsal forebrain. The authors notice the absence of studies modelling mid-brain connectivity but the lines from 30-40 lack references. The authors should discuss here specific animal studies showing how this connectivity is important for overall brain function and also how this pathway is degenerated in PD to bolster the need for MLOs with striatal organoids to gain a human development and disease perspective.

g. P9 Line 21- Insert appropriate references. The citation of the paper should be the place at which the evidence is first described, not at the last.

Minor comments

1. P2, Line 35- remove Development of Midbrain-like Organoids

2. P2, Line 38- Correct “tand” to “and”

3. P11- Line 15- Correct “tcomplex” to “complex”

Associate Editor

Comments to the Author:

Please note the comments made by the reviewers.

Date Sent:

20-Jun-2023

**Author response**

Reviewer: 1

Comments to the Author

In is review, the authors introduced the developing of midbrain-like organoids (MLOs) models, summarized their biological relevance and applications in disease modeling and therapies. Additionally, they discussed the limitations and potential improvements of MLOs technology in the future. Overall, authors do a commendable job in summarizing the state-of-art in the field with considerable details on the protocols and findings. I only have a few comments for the authors to improve their manuscript:

We thank the reviewer for the positive feedback and have made the appropriate amendments according to the suggestions provided by the reviewer as below.

Major point

1. In Figure1, the timeline of unguided v.s. guided organoid protocol is not correct. The protocols developed by Kadoshima et. al., 2013 and Pasca et. al., 2015 used SMAD inhibitor and some growth factors to restrict cell fates and should be considered as guided protocols. Qian et al. developed guided protocol for several region specific brain organoids. It should also include subregions specific organoids, such as Huang et al. 2022 for arcuate nucleus organoid and Kiral et al. 2023 for ventralized thalamic organoids.

We thank the reviewer for highlighting the importance of including subregion-specific organoids protocol in Figure 1. We have now included Huang et al. 2021 [33] and Kiral et al. 2023 [29] as part of Figure 1. Also, we have in-text a more detailed coverage of the available protocols in Section 1.2, paragraph 3.

2. The structure of section 1- Generating Human Dopaminergic Neuronal Models is a little confusion. Clearly, section 1.1- Generating Three-dimensional Brain Organoids does not belong to the topic of section 1. Instead, it should be part or introduction of section 1.2- Development of Midbrain-like Organoids. I suggest that the authors reorganize section 1 by firstly introducing 2D dopaminergic neuronal models and then 3D Midbrain-like Organoids.

We thank the reviewer for pointing out the potential confusion in Section 1.1. Section 1 provides an overview of the development of human dopaminergic neuronal models including the progress from 2D to 3D models. To organise the ideas better, we have now split the contents in Sections 1.1 into Section 1.1 Directed Differentiation of Dopaminergic Neurons and Section 1.2 Generating Three-dimensional Brain Organoids to first introduce 2D models before their 3D counterparts. No changes have been made to the content and flow.

Minor points

1. A typo in Page 2 Line 38 “tand” should be “and”

“tand” edited to “and”

2. Page 3 line 31, the authors may cite the protocol developed by Vannary Tieng as well.

Besides, Matrigel embedding is not a common feature of these protocols since several of them (for example: L. M. Smits et al.; X. Qian et al.; Tieng et al.) didn’t use it. It’d be better to remove the sentence “Matrigel is added to the culture ……” in line 35-36.

We have now included new citation [41] to page 3 line 25. Also, to minimise confusion about the use of Matrigel in MLO protocols, we now emphasise that “In some protocols, extracellular matrices such as Matrigel are added…”(Page 3, lines 28-29).

3. Page 5 line 48-49, the statement that “cerebral organoids derived from hPSCs typically do not contain non-neural lineages” is not true for unguided protocol that can generate microglia, which has also been discussed in this manuscript in section 2.2.2.

We recognise the concern highlighted by the reviewer. To correct our statement, we have modified the sentence that follows to “Thus, key cell types such as mesoderm-derived endothelial cells (ECs) that form the vasculature, and microglia, which are the brain’s resident immune cells are absent in most guided organoid-making protocols.” (Page 6, line 4)

4. Page 10 line 41-42, delete the repeated sentence “were significantly different in patient derived MLOs.”

The repeated section of the sentence have now been removed.

5. Page 15 line 23-24, they mention transplanted organoids as a method to rescue motor and visual function in the review but do not give any references

We apologise for the oversight. The citations [152, 169] have now been added (Page 15, lines 29-30).

Reviewer: 2

Comments to the Author

Review of:

“Midbrain organoids and disease”

In this review, the author(s) summarize the current literature on 2D and 3D models to study midbrain dopaminergic neuronal differentiation and their use in studying neurological disorders such as Parkinson’s disease (PD).

This is a useful review with a detailed and very well-written section 3 summarizing the salient features of various studies using 2D/3D neuronal and organoid models to study PD in vitro. The therapeutic relevance and future scope of midbrain organoids is also well written but Sections 1 and 2 need refinement as detailed below.

We thank the reviewer for the kind comments. Amendments have been made to the review according to the suggestions provided by the reviewer.

Major comments

1. The review title is too generic. To better reflect the authors' focus on the various 2D/3D models of mid-brain and dopaminergic neuronal development and their relevance in studying PD, the title should be revised to incorporate this aspect.

To better incorporate the scope of our review, we have modified the title of to “Midbrain organoids – Development and Applications in Parkinson’s Disease”.

2. The authors have summarized the existing methodologies but the review should include more authors’ own opinions on how newer and more robust protocols can be developed based on existing understanding of midbrain and dopaminergic neuronal development as there are not many studies in this area. Below are summarised various instances in Sections 1 and 2 where the authors should elaborate more:

a. Section 1 and Section 1.2 - The authors summarise the various protocols but do not discuss how the existing 2D/3D protocols mimic neurodevelopment in a detailed manner. A detailed molecular analysis will help identify existing lacunae in the protocols and help pave the way for designing future additions to the existing protocols. Suggestion: A schematic if added to this section will vastly improve the import of the section.

We thank the reviewer for the suggestion. The focus of this review largely revolves around the development and applications of MLOs as such we haven’t discussed in great molecular detail the development of dopaminergic neurons. The molecular details have been reviewed in greater depth in another recent review Yeap et al. 2023. Nonetheless, we have discussed in Section 2.1 of the review the relevance of MLOs in the context of rodents and human dopaminergic neurons. We believe that this is also important for looking at future improvements to be made to MLOs.

b. P3, Line 53- what are these midbrain patterning factors? Discussion on their role would be useful.

We thank the reviewer for highlighting the concern, but we believe that the roles of midbrain patterning factors have been discussed in Section 1.1, paragraph 2. To make clearer the roles of the patterning factors, we have modified the sentence to include “that manipulate the WNT and SHH signalling pathways” (Page 3, lines 43-44).

c. P4, Line 11- The title is too generic /broad. This should be amended to be more specific.

We have structured the section titles into 4 separate themes which broadly cover the topics that will be covered in each section. Nonetheless, we have provided sub-section titles that describe our content in greater detail.

d. P4, Line 13. The entire section is on the various neuronal subtypes in the mouse and human midbrain and not MLOs. This should be amended.

The sub-section title has been modified to “Dopaminergic Neuronal Subtypes in Rodents and Humans”.

e. P5, Lines 21-28 - The authors have summarised the results of Smits et al., and Fiorenzano et. al in a few lines but since the major focus of the review is the development of MLOs and their usage in studying disorders, the review should include a detailed molecular analysis and discussion on how the two studies demonstrate the ability of MLOs to mimic dopaminergic neurogenesis.

We thank the reviewer for the suggestion to include more details about the findings in these studies. We have extended our discussion of their molecular findings as reflected on Page 5, lines 22-23 & 26-29.

f. Section 2.2.3 – Multi-region organoids- a major portion of this section discusses muti-**region** organoids in the dorsal forebrain. The authors notice the absence of studies modelling mid-brain connectivity but the lines from 30-40 lack references. The authors should discuss here specific animal studies showing how this connectivity is important for overall brain function and also how this pathway is degenerated in PD to bolster the need for MLOs with striatal organoids to gain a human development and disease perspective.

We understand the concerns highlighted by the reviewer. To improve the coverage of our review, we have included a discussion of earlier and current studies, including citations [91-99], that discuss the projection of the midbrain to other brain sections (Page 8, lines 31-32, 34-35 & 37-39) to highlight the potential of multi-region organoids in future studies.

g. P9 Line 21- Insert appropriate references. The citation of the paper should be the place at which the evidence is first described, not at the last.

We have now inserted the appropriate references to Page 9, line 27-28.

Minor comments

1. P2, Line 35- remove Development of Midbrain-like Organoids

2. P2, Line 38- Correct “tand” to “and”

3. P11- Line 15- Correct “tcomplex” to “complex”

Minor comments have been amended as suggested by the reviewer.

**Revised Submission - OXFNSC-2023-006.R1**

**Reviewer 1**

Comments to the Author

The manuscript is greatly improved. Here are a few minor points:

1.2. Reference #35 reported region specific organoids for cortex, hypothalamus and midbrain.

2.1 DA neurons: When discussing DA neurons differentiation, it will be nice to include studies transplanting them into money brain and ongoing clinical trails.

2.2. Discussion of astrocytes: One recent study examined the maturation of glia cells in different protocols and it may not take that long to get glia cells with molecular feature in the adult human brain (PMID: 36608676).

2.2.1. Discussion of vascularized organoids: It should be pointed out that even with vascular-like structures in organoids in vitro, there is no active perfusion. Therefore=, impact on survival of core of organoids is limited. There are alternative approaches, such as sliced organoids (PMID: 33328611 and 32142682).

5. Discussion of future midbrain organoids: One the important future goal is to generate brain sub-region specific organoids, such as for hypothalamus (PMID: 33961804) and thalamus (PMID: 37019105). The same should be developed for midbrain, which is a heterogeneous region. There is a need to discuss the limitation of MLO as a developmental model to study age-dependent neurodegeneration in PD and potential approaches to overcome such limitations.

**Reviewer 2**

Comment to the Author

The authors have addressed all my concerns. The manuscript can be accepted for publication.

**Decision letter - OXFNSC-2023-006.R1**

24-Jul-2023

Dear Dr. Sun,

Manuscript ID OXFNSC-2023-006.R1 entitled "Midbrain organoids – Development and Applications in Parkinson’s Disease" which you submitted to the Oxford Open Neuroscience, has been reviewed. The comments of the reviewer(s) are included at the bottom of this letter.

Please note that this journal operates with transparent peer review. This means that if your submission is accepted for publication, the full peer review history of your article will publish online alongside your article. This includes reviewer comments, editor decision letters, and your author responses.

The reviewer(s) have recommended publication, but also suggest some minor revisions to your manuscript. Therefore, I invite you to respond to the reviewer(s)' comments and revise your manuscript.

To revise your manuscript, log into https://mc.manuscriptcentral.com/oxfnsc and enter your Author Centre, where you will find your manuscript title listed under "Manuscripts with Decisions." Under "Actions," click on "Create a Revision." Your manuscript number has been appended to denote a revision.

You may also click the below link to start the revision process (or continue the process if you have already started your revision) for your manuscript. If you use the below link you will not be required to login to ScholarOne Manuscripts.

*** PLEASE NOTE: This is a two-step process. After clicking on the link, you will be directed to a webpage to confirm. ***

https://mc.manuscriptcentral.com/oxfnsc?URL_MASK=266fe31baccf4cac9e55cd215437b3f1

You will be unable to make your revisions on the originally submitted version of the manuscript. Instead, revise your manuscript using a word processing program and save it on your computer. Please also highlight the changes to your manuscript within the document by using the track changes mode in MS Word or by using bold or colored text.

Once the revised manuscript is prepared, you can upload it and submit it through your Author Centre.

When submitting your revised manuscript, you will be able to respond to the comments made by the reviewer(s) in the space provided. You can use this space to document any changes you make to the original manuscript. In order to expedite the processing of the revised manuscript, please be as specific as possible in your response to the reviewer(s).

IMPORTANT: Your original files are available to you when you upload your revised manuscript. Please delete any redundant files before completing the submission.

Because we are trying to facilitate timely publication of manuscripts submitted to the Oxford Open Neuroscience, your revised manuscript should be uploaded as soon as possible. If it is not possible for you to submit your revision in a reasonable amount of time, we may have to consider your paper as a new submission.

Once again, thank you for submitting your manuscript to the Oxford Open Neuroscience and I look forward to receiving your revision.

Sincerely,

Dr. Orly Reiner

Senior Editor, Oxford Open Neuroscience

orly.reiner@weizmann.ac.il, orly.reiner@weizmann.ac.il

Reviewer: 1

Comments to the Author

The manuscript is greatly improved. Here are a few minor points:

1.2. Reference #35 reported region specific organoids for cortex, hypothalamus and midbrain.

2.1 DA neurons: When discussing DA neurons differentiation, it will be nice to include studies transplanting them into money brain and ongoing clinical trails.

2.2. Discussion of astrocytes: One recent study examined the maturation of glia cells in different protocols and it may not take that long to get glia cells with molecular feature in the adult human brain (PMID: 36608676).

2.2.1. Discussion of vascularized organoids: It should be pointed out that even with vascular-like structures in organoids in vitro, there is no active perfusion. Therefore=, impact on survival of core of organoids is limited. There are alternative approaches, such as sliced organoids (PMID: 33328611 and 32142682).

5. Discussion of future midbrain organoids: One the important future goal is to generate brain sub-region specific organoids, such as for hypothalamus (PMID: 33961804) and thalamus (PMID: 37019105). The same should be developed for midbrain, which is a heterogeneous region. There is a need to discuss the limitation of MLO as a developmental model to study age-dependent neurodegeneration in PD and potential approaches to overcome such limitations.

Reviewer: 2

Comments to the Author

The authors have addressed all my concerns. The manuscript can be accepted for publication

Associate Editor

Comments to the Author:

(There are no comments.)

Date Sent:

24-Jul-2023

**Author response**

We thanks the reviewers for their helpful comments. Reviewer 2 is Ok to accept our manuscript for publication while reviewer 1 raised a few minor issues. Below please find our response to reviewer 1's suggestions.

Reviewer: 1

Comments to the Author

The manuscript is greatly improved. Here are a few minor points:

1.2. Reference #35 reported region specific organoids for cortex, hypothalamus and midbrain.

We thank the reviewer for highlighting that. We’ve now also cited Reference #35 (now reference #28) for the making of cortex, hypothalamus and midbrain organoids.

2.1 DA neurons: When discussing DA neurons differentiation, it will be nice to include studies transplanting them into money brain and ongoing clinical trails.

We thank the reviewer for the suggestion. Transplantations of DA neurons or MLOs have already been mentioned and discussed when highlighting the pathological and therapeutic relevance of MLOs in Section 5. As this allows us to discuss the possibilities of MLO transplantation in a broader context, we prefer to keep this part in section 5 instead of section 2.1 DA neuron differentiation.

2.2. Discussion of astrocytes: One recent study examined the maturation of glia cells in different protocols and it may not take that long to get glia cells with molecular feature in the adult human brain (PMID: 36608676).

We acknowledge that there are protocols that suggest the production of glia cells with transcriptomic signatures similar to that of human glial samples and have highlighted that in Section 2.2, Page 5 (lines 40-44 in the revised manuscript) with the addition of References #57-61. It is still generally recognised that glial populations in organoids take an extended culture period to appear and are functionally immature in many aspects as compared to their counterparts in adult brains . As such, we would prefer to hold back on making comments suggesting that mature glia can be generated in organoids in shorter timeframes, which is also relative to the experimental timeframe of individual groups.

2.2.1. Discussion of vascularized organoids: It should be pointed out that even with vascular-like structures in organoids in vitro, there is no active perfusion. Therefore=, impact on survival of core of organoids is limited. There are alternative approaches, such as sliced organoids (PMID: 33328611 and 32142682).

We thank the reviewer for highlighting the current limitation of vascularised organoids. Accordingly, we have added a few lines to Section 2.2.1, Page 7 (lines 9-12 of the revised version) to highlight the limitation as well as potential strategies for consideration to overcome the limitation. References #71 and #72 were also added to complete the section.

5. Discussion of future midbrain organoids: One the important future goal is to generate brain sub-region specific organoids, such as for hypothalamus (PMID: 33961804) and thalamus (PMID: 37019105). The same should be developed for midbrain, which is a heterogeneous region. There is a need to discuss the limitation of MLO as a developmental model to study age-dependent neurodegeneration in PD and potential approaches to overcome such limitations.

We recognise the limitation of MLO as a developmental model in the studying of age-dependent neurodegeneration. As such, we have discussed in Section 5 under Pathological relevance the challenges of using current MLO models for studying adult-onset diseases. As part of our discussion, we also suggested possible ways that may be tested to achieve accelerated aging in MLOs.

**Revised Submission - OXFNSC-2023-006.R2**

**Reviewer 1**

The authors have largely addressed my comments.

**Decision letter - OXFNSC-2023-006.R2**

08-Aug-2023

Dear Dr. Sun,

It is a pleasure to accept your revised manuscript entitled "Midbrain organoids – Development and Applications in Parkinson’s Disease" in its current form for publication in the Oxford Open Neuroscience. The comments of the reviewer(s) who reviewed your manuscript are included at the foot of this letter.

Please note that this journal operates with transparent peer review. This means that the full peer review history of your article will publish online alongside your article. This includes reviewer comments, editor decision letters, and your author responses.

Next steps

You will receive an email from no-reply@scipris.com within roughly one week. This is your invitation to sign up for an account with SciPris, Oxford University Press’ author portal hosted by Aptara. You will need to create an account if you do not already hold one. Please register or log into your account and follow the online instructions which will guide you through signing your licence and paying the APC. The email and the portal have clearly signposted support options if you need any help during this process.

Please note that SciPris is a completely different system from ScholarOne, so your credentials to submit your manuscript here will not work there. Once you’ve created a SciPris account, you will be able to use it whenever you publish with Oxford Open Neuroscience or any OUP journal. Please note that OUP will only ever request payment for applicable fees be made via SciPris or to an OUP bank account. If you ever have concerns about the legitimacy of a request, please do not hesitate to contact a customer services agent via the SciPris portal or directly via oupsupport@scipris.com.

Thank you for your fine contribution. On behalf of the Editors of the Oxford Open Neuroscience, we look forward to your continued contributions to the Journal.

Sincerely,

Dr. Orly Reiner

Senior Editor, Oxford Open Neuroscience

orly.reiner@weizmann.ac.il, orly.reiner@weizmann.ac.il

Reviewer: 1

Comments to the Author

The authors have largely addressed my comments.

Associate Editor

Comments to the Author:

(There are no comments.)

Date Sent:

08-Aug-2023
